# ﻿Four new species of *Collybiopsis* (Omphalotaceae, Agaricales) from subtropical regions of China

**DOI:** 10.3897/mycokeys.118.155560

**Published:** 2025-06-11

**Authors:** Ling Ding, Ben-Jian Zhong, Hui Zeng, Sheng-Nan Wang, Jun-Qing Yan

**Affiliations:** 1 Jiangxi Provincial Key Laboratory of Excavation and Utilization of Agricultural Microorganisms, Jiangxi Agricultural University, Nanchang 330045, China; 2 Institute of Edible mushroom, Fujian Academy of Agricultural Sciences, Fuzhou 350011, China; 3 Jiangxi Provincial Key Laboratory of Subtropical Forest Resource Cultivation, College of Forestry, Jiangxi Agricultural University, Nanchang 330045, China

**Keywords:** Basidiomycetes, new taxa, phylogeny, taxonomy

## Abstract

Four new species of *Collybiopsis*, namely *C.subpolygramma*, *C.fucata*, *C.dentata*, and *C.latispora*, were discovered in the subtropical regions of China. These species were identified based on morphological characteristics and molecular analysis. Morphologically, *C.subpolygramma* is characterized by its pileal surface, white in color with a light brown center; slender, cylindrical cheilocystidia; and absence of pleurocystidia. *C.fucata* is recognized by the reddish-brown, hygrophanous pileus; adnate to adnexed lamellae; and relatively broad basidiospores. *C.dentata* is identified by free lamellae, fusiform to utriform pleurocystidia, and relatively broad basidiospores. *C.latispora* is distinct by relatively broad basidiospores and a minute, snow-white ring or ruff at the stipe base. In the phylogenetic analysis based on combined ITS and LSU sequences, the four new species formed distinct and stable branches (BI-PP = 1; ML-BP = 100%), respectively. *Collybiopsissubpolygramma* formed a sister lineage with *C.polygramma*. The new species *C.fucata*, *C.dentata*, and *C.latispora* were found to be nested within the *C.ramealis* species complex clade. Detailed descriptions, color photos, and a key to related species are presented.

## ﻿Introduction

*Collybiopsis* (J. Schröt.) Earle was established by Earle in 1909, with *C.ramealis* (Bull.) Millsp. designated as the type species (Petersen and Hughes 2021; Kim et al. 2022). This genus includes 91 records in Index Fungorum [https://indexfungorum.org (accessed on 12 May 2025)], comprising approximately 84 species. The species of *Collybiopsis* are characterized by collybioid, gymnopoid, marasmielloid, omphalioid, and pleurotoid basidiomata; free to decurrent lamellae; a central to eccentric, insititious to subinsititious stipe; ellipsoid to oblong, inamyloid, and hyaline basidiospores with white spore prints; presence of caulocystidia; and coralloid or diverticulate terminal elements of pileipellis (Oliveira et al. 2019; Kim et al. 2022; Razzaq et al. 2023). Species of *Collybiopsis* are mainly distributed in tropical and temperate climates, with most species being saprobic on dead wood of angiosperms and gymnosperms, as well as leaf litter (Petersen and Hughes 2021; Kim et al. 2022; Razzaq et al. 2023). Some species of *Collybiopsis*, as members of wood-decaying fungi, play a crucial role in maintaining the balance of forest ecosystems and in degradation and reduction processes. For instance, they secrete various bio-enzymes, such as extracellular enzymes, to degrade the cellulose, lignin, and hemicellulose in wood, thereby promoting the material cycle in the ecosystem (Wei and Dai 2004; Cho et al. 2024).

Previously, species within *Collybiopsis* were included within *Collybia* s. l. (Petersen and Hughes 2024). With the advancement of phylogenetic studies on *Collybia* s. l., the concept of *Collybia* s. s. was redefined, and the remaining taxa were transferred to *Collybiopsis*, *Gymnopus* (Pers.) Gray, and *Marasmiellus* Murrill (Oliveira et al. 2019; Razzaq et al. 2023; Petersen and Hughes 2024). However, both *Gymnopus* and *Marasmiellus* are polyphyletic in the phylogenetic analysis (Oliveira et al. 2019). Currently, the redefined *Collybiopsis* includes species of *Collybiopsis*, some species of Gymnopussect.Vestipedes, and *Marasmiellus* (Petersen and Hughes 2021; Kim et al. 2022; Razzaq et al. 2023).

In China, 25 species of *Collybiopsis* have been reported, including nine new species described from Yunnan and Guangdong provinces and Guangxi and Xizang Autonomous Regions (Zhang et al. 2023b; Li et al. 2024; Liu et al. 2024; Liu et al. 2025a). During surveys in the subtropical regions of China, several species with characteristics of *Collybiopsis* were found that do not correspond to any described species. Based on morphological comparisons and phylogenetic analyses, four of these species were further confirmed as new species of *Collybiopsis*.

## ﻿Materials and methods

### ﻿Morphological study

Specimens are deposited in the Herbarium of Fungi, Jiangxi Agricultural University (HFJAU). The specimens were collected from 2021 to 2024 and stored in dried condition. Macroscopic characteristics were recorded from fresh specimens. The color codes were referenced from the Methuen Handbook of Colour (Kornerup and Wanscher 1978). Micromorphological structures were observed and measured under an Olympus BX53 microscope (Olympus Corporation, Tokyo, Japan) by making squash preparations of sections of dried specimens that were placed in 5% KOH solution or H_2_O, and 1% Congo red was used as the staining agent for observing colorless tissues. Melzer’s reagent was selected for determining whether the spores were amyloid or not (Horak 2005; Chen et al. 2024). At least 20 basidiospores, basidia, and cystidia were measured for each collection. The range of spore size is expressed in the format (a) b–c (d), where “a” and “d” represent the minimum and maximum values, and 90% of the spores fall within the range “b–c”. The basidiospore quotient (Q = length / width) was calculated based on the measurements of basidiospores. The meanings of the other spore characteristics are as follows: “av” symbolizes average value; “n” means the number of measured spores; and “Qm” indicates the standard deviation of “Q” (Bas 1969; Chen et al. 2024).

### ﻿DNA extraction, PCR amplification, and sequencing

Genomic DNA was extracted from dried specimens using the NuClean Plant Genomic DNA kit (CWBIO, China). The ITS and LSU regions were amplified separately using the primer pairs of ITS1F/ITS4 (White et al. 1990; Gardes and Bruns 1993) and LR0R/LR5 or LR7, respectively (Vilgalys and Hester 1990).

PCR amplification was conducted using a 25 µL reaction system as follows: 1 µL of DNA, 1 µL of each primer, 9.5 µL of ddH_2_O, and 12.5 µL of 2× TaqMaster Mix [Qing Ke Biotechnology Co. Ltd. (Wuhan City, China)]. PCR was performed using a touchdown program for all regions: initial 95 °C for 5 min, and then 14 cycles of denaturing at 95 °C for 30 s, annealing at 65 °C for 45 s (with a decrease of 1 °C per cycle), extension at 72 °C for 1 min, and then 30 cycles of denaturing at 95 °C for 30 s, annealing at 52 °C for 30 s, and extension at 72 °C for 1 min, with the final extension at 72 °C for 10 min (Bau and Yan 2021; Chen et al. 2024). The PCR products were sequenced by Qing Ke Biotechnology Co. Ltd. (Wuhan City, China).

### ﻿Alignment and phylogenetic analyses

In total, 255 sequences (156 ITS sequences and 99 LSU sequences) of 156 samples were used for phylogenetic analyses based on ML and BI. The selection of sequences for the phylogenetic analyses was based on the results of ITS BLAST and studies by Petersen and Hughes (2021) and Kim et al. (2022) (Table 1). Following the research of Kim et al. (2022), *Rhodocollybiabutyracea* (Bull.) Lennox and *R.maculata* (Alb. & Schwein.) Singer were selected as outgroups.

**Table 1.** A list of species and sequences used in the phylogenetic analyses. Newly generated sequences are in bold.

Species	Location	Voucher Number	GenBank Number		Sequence reference
			ITS	LSU	
*Collybiopsisaffixa*	Australia	FT0004B	PP508259	–	Unpublished in GenBank
*Collybiopsisalbicantipes*	Korea	SFC20170725-35 Holotype	OL467272	OL462811	Kim et al. (2022)
*Collybiopsisalbicantipes*	Korea	SFC20180704-86	OL467273	OL462812	Kim et al. (2022)
*Collybiopsisalpina*	China	HMJAU 60410 Holotype	PP151538	PP151568	Li et al. (2024)
*Collybiopsisalpina*	China	HMJAU 60411	PP151537	–	Li et al. (2024)
*Collybiopsisattenuata*	China	HMAS296736 Holotype	PP741392	–	Unpublished in GenBank
*Collybiopsisbaiyunensis*	China	GDGM93885 Holotype	OR598795	OR598808	Liu et al. (2024)
*Collybiopsisbaiyunensis*	China	GDGM93886	OR598792	OR598809	Liu et al. (2024)
*Collybiopsisbambusicola*	China	BJFC 032412 Holotype	MW969675	ON697204	Zhang et al. (2023b)
*Collybiopsisbiformis*	USA	TFB14251	KJ416245	KJ189567	Petersen and Hughes (2014)
*Collybiopsisbiformis*	USA	TFB14250	KJ416246	KJ189568	Petersen and Hughes (2014)
*Collybiopsisbiformis*	USA	TFB13814	KJ416249	KJ189569	Petersen and Hughes (2014)
*Collybiopsisbiformis*	USA	TFB13890	KJ416248	KJ189570	Petersen and Hughes (2014)
*Collybiopsisbillbowesii*	Africa	SFSU DED 8250 Holotype	MF100989	–	Desjardin and Perry (2017)
*Collybiopsisbrunneigracilis*	Java/Bali	AWW01	AY263434	AY639412	Unpublished in GenBank
*Collybiopsiscalifornica*	Canada	TENN-F-052617	MN413338	–	Petersen and Hughes (2021)
*Collybiopsiscarneopallida*	Italy	BRNM 747442	OM522632	–	Antonín et al. (2022)
*Collybiopsiscimrmanii*	Portugal	BRNM 828679 Holotype	MW924062	OM333232	Unpublished in GenBank
*Collybiopsiscimrmanii*	Portugal	BRNM 828680	MW924061	OM333231	Unpublished in GenBank
*Collybiopsisclavicystidiata*	Korea	SFC20180705-26	OL467250	OL462816	Kim et al. (2022)
*Collybiopsisclavicystidiata*	Korea	SFC20180705-84 Holotype	OL467252	OL462817	Kim et al. (2022)
*Collybiopsisclavicystidiata*	Korea	SFC20180713-09	OL467251	OL462819	Kim et al. (2022)
*Collybiopsiscomplicata*	USA	TENN-F-055766 Holotype	DQ450029	–	Mata et al. (2007)
*Collybiopsiscomplicata*	USA	TENN-F-065811	OR500517	OR500517	Unpublished in GenBank
*Collybiopsisconfluens*	Canada	TFB14409 Holotype	KP710278	KJ189585	Hughes and Petersen (2015)
*Collybiopsisconfluens*	Canada	TFB14389	KP710279	KJ189584	Hughes and Petersen (2015)
*Collybiopsisconfluens*	USA	TFB14075	KP710281	KJ189581	Hughes and Petersen (2015)
*Collybiopsiscylindrica*	Costa Rica	TENN 058097 Holotype	NR_119464	–	Schoch et al. (2014)
***Collybiopsisdentata***	**China**	**HFJAU2586 Holotype**	**PV424096**	–	**This work**
***Collybiopsisdentata***	**China**	**HFJAU5718**	**PV424098**	–	**This work**
*Collybiopsisdiaphana*	Mexico	Cesar202 Holotype	MT232390	–	César et al. (2020)
*Collybiopsisdichroa*	USA	TFB9623	MW396865	MW396865	Petersen and Hughes (2021)
*Collybiopsisdisjuncta*	USA	TENN 69172 Holotype	NR_137865	–	Petersen and Hughes (2014)
*Collybiopsisdisjuncta*	USA	TFB14281	KJ416253	KY019643	Petersen and Hughes (2014)
*Collybiopsiseneficola*	Canada	TENN 69123 Holotype	NR_137613	NG_059502	Petersen et al. (2014)
*Collybiopsiseneficola*	Canada	100921AV04	KJ128265	KJ189590	Petersen et al. (2014)
*Collybiopsiseneficola*	Alaska	MICH PK6975	KP710270	KP710304	Hughes and Petersen (2015)
*Collybiopsisfilamentipes*	USA	TFB13962 Holotype	MN897832	MN897832	Petersen and Hughes (2021)
*Collybiopsisfoliiphila*	India	CUH AM090 Holotype	NR_154176	NG_060320	Dutta et al. (2015)
*Collybiopsisfoliiphila*	India	CUM AM101	KP317638	KP317636	Dutta et al. (2015)
***Collybiopsisfucata***	**China**	**HFJAU2535 Holotype**	**PV424094**	–	**This work**
***Collybiopsisfucata***	**China**	**HFJAU5343**	**PV424095**	**PV366624**	**This work**
*Collybiopsisfulva*	Korea	KA13-0216	OL467257	OL462793	Kim et al. (2022)
*Collybiopsisfulva*	Korea	KA13-0333	OL467258	OL462794	Kim et al. (2022)
*Collybiopsisfulva*	Korea	KA15-0210 Holotype	OL467259	OL462795	Kim et al. (2022)
*Collybiopsisfurtiva*	USA	TENN-F-051097	MN413343	MW396879	Petersen and Hughes (2021)
*Collybiopsisgibbosa*	Australia	MEL 2382838	KP012713	–	Unpublished in GenBank
*Collybiopsisgibbosa*	Brazil	URM 90012	KY061202	KY061202	Unpublished in GenBank
*Collybiopsisgibbosa*	Brazil	URM 90006	KY061203	KY061203	Unpublished in GenBank
*Collybiopsishasanskyensis*	Russia	TFB11846	MN897829	–	Petersen and Hughes (2021)
*Collybiopsishirtelloides*	Africa	SFSU DED 8318 Holotype	MF100975	–	Desjardin and Perry (2017)
*Collybiopsishumillima*	unknown	DA-22 029	OQ850983	–	Unpublished in GenBank
*Collybiopsisincarnata*	China	Liu 1236 Holotype	PQ638405	PQ637004	Liu et al. (2025a)
*Collybiopsisincarnata*	China	Liu 1240	PQ638406	PQ637005	Liu et al. (2025a)
*Collybiopsisindoctus*	Java/Bali	AWW04	AY263439	–	Unpublished in GenBank
*Collybiopsisistanbulensis*	Turkey	KATO Fungi 3596	KX184795	KX184796	Sesli et al. (2018)
*Collybiopsisjuniperina*	USA	TFB9889	AY256708	KY019637	Mata et al. (2004)
*Collybiopsisjuniperina*	Argentina	TFB10782	KY026661	KY026661	Petersen and Hughes (2016)
*Collybiopsiskoreana*	Korea	SFC20120821-84	OL467269	OL546545	Kim et al. (2022)
*Collybiopsiskoreana*	Korea	SFC20150721-10	OL467271	OL462802	Kim et al. (2022)
***Collybiopsislatispora***	**China**	**HFJAU3109 Holotype**	**PV424097**	**PV366626**	**This work**
*Collybiopsisluxurians*	Korea	NIBRFG0000502888	OL467248	OL462803	Kim et al. (2022)
*Collybiopsisluxurians*	Korea	SFC20190731-18	OL467249	OL462804	Kim et al. (2022)
*Collybiopsisluxurians*	USA	TFB10350	AY256709	AY256709	Mata et al. (2004)
*Collybiopsisluxurians*	Sweden	TFB4283-10	KJ416240	–	Petersen and Hughes (2014)
*Collybiopsisluxurians*	USA	TFB9121	KY026649	KY026649	Petersen and Hughes (2016)
*Collybiopsisluxurians*	USA	TFB14060	MW396871	MW396871	Petersen and Hughes (2021)
*Collybiopsismedogensis*	China	Liu 1048	PQ638400	PQ636999	Liu et al. (2025a)
*Collybiopsismedogensis*	China	Liu 1055 Holotype	PQ638399	PQ636998	Liu et al. (2025a)
*Collybiopsismelanopus*	Java/Bali	AWW54 Holotype	NR_137539	NG_060624	Unpublished in GenBank
*Collybiopsismelanopus*	India	CUH AM093	KM896875	–	Dutta et al. (2015)
*Collybiopsismenehune*	USA	AWW15	AY263443	AY639424	Unpublished in GenBank
*Collybiopsismesoamericana*	Costa Rica	TFB11005	DQ450035	KY019632	Mata et al. (2007)
*Collybiopsismesoamericana*	Costa Rica	TFB10411	DQ450036	–	Mata et al. (2007)
*Collybiopsismesoamericana*	Costa Rica	REH7379	AF505768	–	Mata et al. (2007)
*Collybiopsismicromphaleoides*	USA	TENN 68165 Holotype	NR_137864	NG_059734	Petersen and Hughes (2014)
*Collybiopsismicromphaleoides*	USA	TFB14282	KJ416243	KY019645	Petersen and Hughes (2014)
*Collybiopsisminor*	USA	TENN-F-059993	MN413334	MW396880	Petersen and Hughes (2021)
*Collybiopsisminor*	USA	TFB5434	MW396872	MW396872	Petersen and Hughes (2021)
*Collybiopsisminor*	USA	TFB6284	MW405778	–	Petersen and Hughes (2021)
*Collybiopsismustachia*	Africa	SFSU BAP 670 Holotype	MF100987	–	Desjardin and Perry (2017)
*Collybiopsisneotropica*	Costa Rica	TFB10416	AF505769	–	Unpublished in GenBank
*Collybiopsisnonnulla*	USA	TFB14492	MW396873	MW396873	Petersen and Hughes (2021)
*Collybiopsisnonnulla*	USA	TFB14278	KY026701	KY026701	Petersen and Hughes (2016)
*Collybiopsisobscuroides*	Norway	GB-0150514	KX958399	KX958399	Unpublished in GenBank
*Collybiopsisocella*	Africa	SFSU DED 8280 Holotype	MF100976	–	Desjardin and Perry (2017)
*Collybiopsisomphalodes*	Costa Rica	TFB 10427	DQ450011	–	Mata et al. (2007)
*Collybiopsisomphalodes*	Costa Rica	TENN56734	AY256700	–	Mata et al. (2004)
*Collybiopsisorientisubnuda*	Korea	NIBRFG0000500990 Holotype	OL467262	–	Kim et al. (2022)
*Collybiopsisorientisubnuda*	Korea	SFC20170823-39	OL467263	–	Kim et al. (2022)
*Collybiopsisorientisubnuda*	Korea	SFC20180830-29	OL467264	–	Kim et al. (2022)
*Collybiopsispakistanica*	Pakistan	LAH 37522 Holotype	OP199106	OP209954	Razzaq et al. (2023)
*Collybiopsisparvula*	Costa Rica	TFB10419 Holotype	DQ450060	–	Mata et al. (2007)
*Collybiopsisparvula*	Costa Rica	TFB10421	DQ450061	–	Mata et al. (2007)
*Collybiopsisparvula*	Costa Rica	TFB10425	DQ450062	–	Mata et al. (2007)
*Collybiopsisparvula*	Costa Rica	TFB10422	AF505774	–	Mata et al. (2007)
*Collybiopsisperonata*	Belgium	TFB13743	KY026677	KY026677	Petersen and Hughes (2016)
*Collybiopsisperonata*	Russia	LE-Bin1364	KY026755	KY026755	Petersen and Hughes (2016)
*Collybiopsisperonata*	unknown	CBS 223.37	MH855896	MH867405	Vu et al. (2019)
*Collybiopsisperonata*	Canada	UBCF28402	KP454027	–	Unpublished in GenBank
*Collybiopsispleurocystidiata*	Africa	SFSU BAP 651 Holotype	MF100977	–	Desjardin and Perry (2017)
*Collybiopsispolygramma*	Brazil	URM 90015	KY074640	KY088275	Unpublished in GenBank
*Collybiopsispolygramma*	Puerto Rico	PR2542TN	AY842954	–	López-Ferrer (2004)
*Collybiopsispolygramma*	Brazil	URM 90016	KY074641	KY088274	Unpublished in GenBank
*Collybiopsisprolapsis*	USA	TENN-F-051101	MW396874	MW396874	Petersen and Hughes (2021)
*Collybiopsispseudoluxurians*	Costa Rica	REH7348	AF505762	–	Mata et al. (2007)
*Collybiopsispseudoluxurians*	Puerto Rico	PR24TN	AY842957	–	López-Ferrer (2004)
*Collybiopsisquercophila*	USA	TFB14615	KY026736	KY026736	Petersen and Hughes (2016)
*Collybiopsisquercophila*	USA	SFSU25220	KY026761	KY026761	Petersen and Hughes (2016)
*Collybiopsisramealis*	Belgium	TENN-F-065145 Epitype	MN413345	MN413345	Petersen and Hughes (2021)
*Collybiopsisramealis*	Sweden	TFB4727	DQ450030	–	Mata et al. (2007)
*Collybiopsisramealis*	Belgium	TFB13755	KJ416235	KJ189566	Petersen and Hughes (2014)
*Collybiopsisramealis*	Belgium	TENN-F-065146	MN413346	MW396882	Petersen and Hughes (2021)
Collybiopsiscf.ramealis	Korea	SFC20180829-20	OL467261	OL546548	Kim et al. (2022)
*Collybiopsisramulicola*	China	GDGM 43884	KU057798	–	Deng et al. (2016)
*Collybiopsisramulicola*	China	GDGM 44256	KU321529	–	Deng et al. (2016)
*Collybiopsisramulicola*	China	GDGM 50060	KU321530	–	Deng et al. (2016)
*Collybiopsisreadiae*	New Zealand	TFB7571	DQ450034	–	Mata et al. (2007)
*Collybiopsisreadiae*	New Zealand	PDD95844	HQ533036	–	Unpublished in GenBank
*Collybiopsisreadiae*	New Zealand	TFB13056	KJ416244	–	Petersen and Hughes (2014)
*Collybiopsisrodhallii*	Africa	SFSU BAP 627 Holotype	MF100982	–	Desjardin and Perry (2017)
*Collybiopsissalmonea*	China	Liu 1187 Holotype	PQ638404	PQ637003	Liu et al. (2025a)
*Collybiopsissalmonea*	China	Liu 1166	PQ638401	PQ637000	Liu et al. (2025a)
*Collybiopsisschizophylloides*	China	HMJAU 60446 Holotype	PP133256	PP133257	Li et al. (2024)
*Collybiopsisstenophylla*	USA	TENN-F-065943	MN413331	MW396886	Petersen and Hughes (2021)
*Collybiopsisstenophylla*	USA	TENN-F-051099	MN413330	MW396887	Petersen and Hughes (2021)
*Collybiopsisstenophylla*	USA	TFB11558	DQ450032	–	Mata et al. (2007)
*Collybiopsissubcyathiformis*	Puerto Rico	TFB9629	DQ450041	–	Mata et al. (2007)
*Collybiopsissubcyathiformis*	Brazil	URM 90023	KY404982	KY404982	Unpublished in GenBank
*Collybiopsissubcyathiformis*	Brazil	URM 90022	KY404983	KY404983	Unpublished in GenBank
*Collybiopsissubmenehune*	China	Liu 725 Holotype	PQ638396	PQ636995	Liu et al. (2025a)
*Collybiopsissubnuda*	USA	TFB12577	KY026667	–	Petersen and Hughes (2016)
*Collybiopsissubnuda*	USA	WRW 08-462	KY026765	KY026765	Petersen and Hughes (2016)
*Collybiopsissubnuda*	USA	TFB14043	MW396876	MW396876	Petersen and Hughes (2021)
*Collybiopsissubpruinosus*	Portugal	BRNM781138	MK646034	–	Unpublished in GenBank
*Collybiopsissubpruinosus*	USA	TFB11063	DQ450025	–	Mata et al. (2007)
***Collybiopsissubpolygramma***	**China**	**HFJAU2658 Holotype**	**PV424091**	–	**This work**
***Collybiopsissubpolygramma***	**China**	**HFJAU3277**	**PV424092**	**PV366625**	**This work**
***Collybiopsissubpolygramma***	**China**	**HFJAU3551**	**PV424093**	–	**This work**
*Collybiopsissubumbilicata*	Korea	SFC20120802-03	OL467231	OL462786	Kim et al. (2022)
*Collybiopsissubumbilicata*	Korea	SFC20140701-03 Holotype	OL467232	OL462787	Kim et al. (2022)
*Collybiopsissubumbilicata*	Korea	SFC20150902-50	OL467234	OL546540	Kim et al. (2022)
*Collybiopsistayloriae*	Australia	BRIP 76156a Holotype	PP707900	PP707919	Unpublished in GenBank
*Collybiopsistermiticola*	Java and Bali	AWW106	AY263451	AY639430	Unpublished in GenBank
*Collybiopsistrogioides*	Indonesia	AWW51	AY263428	AY639431	Unpublished in GenBank
*Collybiopsisundulata*	Korea	SFC20130808-08	OL467240	OL462814	Kim et al. (2022)
*Collybiopsisundulata*	Korea	SFC20150813-04	OL467241	OL462815	Kim et al. (2022)
*Collybiopsisutriformis*	USA	TFB14334	KY026708	KY026708	Petersen and Hughes (2016)
*Collybiopsisutriformis*	USA	WRW05-1170	KY026764	KY026764	Petersen and Hughes (2016)
*Collybiopsisvaillantii*	USA	TENN–F–065115	KY026676	KY026676	Petersen and Hughes (2016)
*Collybiopsisvellerea*	Korea	NIBRFG0000502858	OL467265	OL462791	Kim et al. (2022)
*Collybiopsisvellerea*	Korea	SFC20120708-02	OL467266	OL462809	Kim et al. (2022)
*Collybiopsisvillosipes*	USA	TFB9539	DQ450058	–	Mata et al. (2007)
*Collybiopsisvillosipes*	New Zealand	TFB12836	KJ416255	–	Petersen and Hughes (2014)
**Outgroup**					
*Rhodocollybiabutyracea*	Canada	TFB 14382	KY026716	KY026716	Petersen and Hughes (2016)
*Rhodocollybiamaculata*	USA	TFB 13989	KY026688	KY026688	Petersen and Hughes (2016)

**Figure 1.**
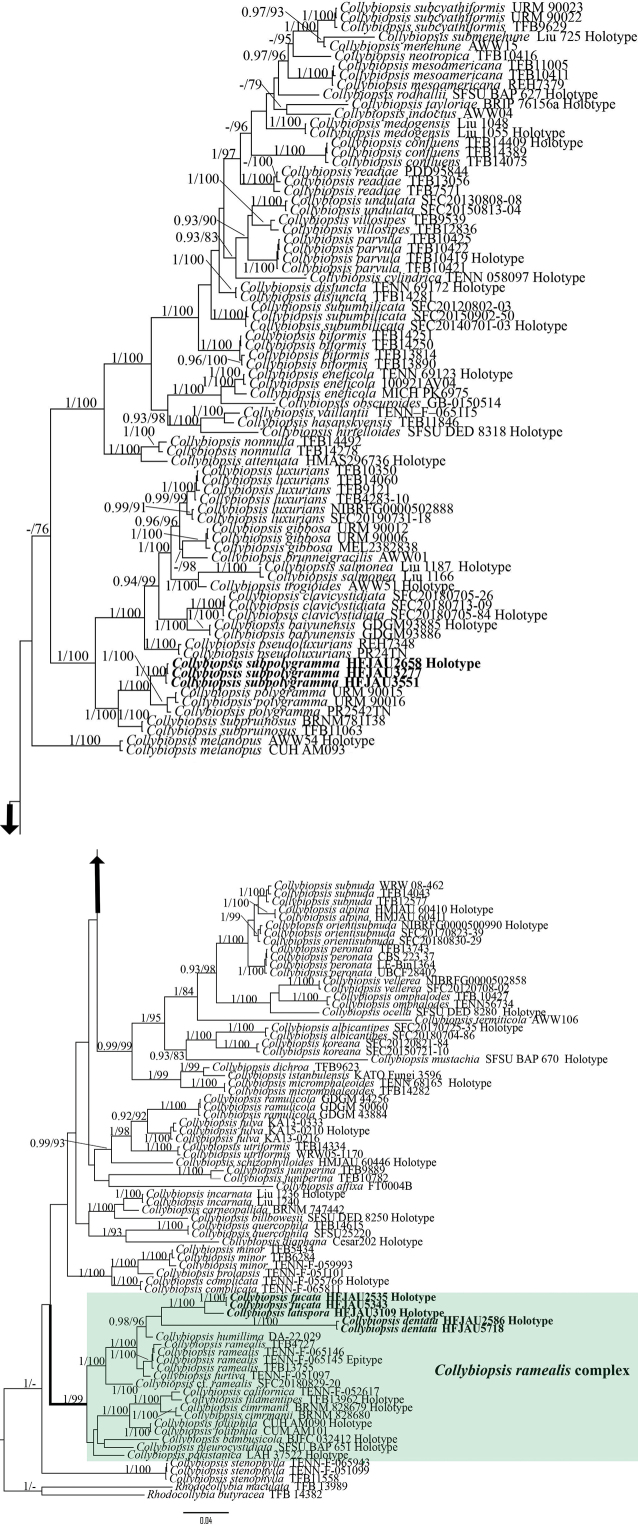
Phylogram of *Collybiopsis* generated by Bayesian inference (BI) analysis based on ITS and LSU, with *Rhodocollybia* spp. designated as the outgroup. Bayesian posterior probabilities (BI-PP) ≥ 0.95 and maximum likelihood bootstrap proportions (ML-BP) ≥ 75 are indicated as PP/BP. New taxa are marked in bold.

The ITS and LSU sequence datasets were separately aligned on the MAFFT online server (Katoh et al. 2019). The processed sequences were subjected to BI and ML phylogenetic analyses using MrBayes v.3.2.7a and IQ-Tree v.2.1.2, respectively (Nguyen et al. 2015). The best-fit models for ML and BI were determined by PartitionFinder (Kalyaanamoorthy et al. 2017), according to the Corrected Akaike Information Criterion (AICc). For the ML analysis, 5,000 replicates were performed based on the ultrafast bootstrap option of ML, which allowed partitions from different seeds. For the BI analysis, the four Monte Carlo Markov chains were set to run for one billion generations, with the computation terminating when the average standard deviation of split frequencies was less than 0.01. The first 25% of the trees were discarded as burn-in. Branches with Bayesian posterior probability (BI-PP) ≥ 0.95 and ML bootstrap support (ML-BP) ≥ 75% are shown in the tree (Fig. 1). All alignments for phylogenetic analyses and the resulting trees were deposited in TreeBASE (ID: 32185, http://purl.org/phylo/treebase/phylows/study/TB2:S32185?x-access-code=30c9f648f6ffbf8543353756fb3d5ef4&format=html).

## ﻿Results

### ﻿Phylogenetic analysis

For the phylogenetic analyses, a total of 2,103 characters were used in the analyses, of which 1,249 were constant, 708 were parsimony-informative, and 146 were singleton. The best-fit models used for the phylogenetic analyses were as follows: GTR+F+I+G4 for ITS and HKY+F+I+G4 for LSU. The log-likelihood value of the ML consensus tree was –20,599.090. And the average standard deviation of split frequencies was < 0.01 after 3,350,000 generations in the BI analysis.

As shown in the phylogenetic tree in Fig. 1, four new species each formed a well-supported monophyletic lineage (BI-PP = 1; ML-BP = 100%). *Collybiopsissubpolygramma* formed a sister lineage with *C.polygramma* (Mont.) R.H. Petersen (BI-PP = 1, ML-BP = 100%). *Collybiopsisfucata*, *C.latispora*, and *C.dentata* were all nested within the *C.ramealis* complex. Among them, *C.fucata* formed a sister lineage with *C.latispora* (BI-PP = 1, ML-BP = 100%). *Collybiopsisdentata* formed a stable branch on its own (BI-PP = 1, ML-BP = 100%).

### ﻿Taxonomy

#### ﻿ Collybiopsis subpolygramma

Taxon classificationFungiAgaricalesOmphalotaceae

J.Q. Yan, L. Ding, & S.N. Wang sp. nov.

D46E8C6F-5864-5559-9857-0F1221ADD180

858728

Fig. 2

##### Etymology.

Referring to its similarity to *C. polygramma*, that pileus is sulcate.

##### Holotype.

China • Zhejiang Province, Lishui City, Qingtian County, Lanni Lake, 1 August 2021, Jun-Qing Yan, Bin-Rong Ke and Zhi-Heng Zeng, HFJAU2658.

##### Diagnosis.

This species is characterized by its pileal surface, white in color with a light brown center; crowded and occasionally forked lamellae; white to black-brown stipe; slender cylindrical cheilocystidia; basidia mainly 2-spored; and absence of pleurocystidia.

##### Macrostructures.

Pileus 32–61 mm in diam., convex to plane, becoming uplifted in age, umbonate at disc, surface smooth, sulcate up to center from the margin, light brown (7D5–6) at the center, gradually becoming lighter towards the margin, margin white. Lamellae adnexed, occasionally forked, crowded, with 1–3 types of lamellulae, white. Stipe 49–68 × 2.4–4.2 mm, central, hollow, cylindrical, equal, white to black-brown (8F5), surface covered with white fibrils, base covered with white mycelium.

##### Microstructures.

Basidiospores in side view [n = 115] (5.9)6.4–8.1(8.7) × (3.7)4.1–5.1(5.2) µm (av = 7.2 × 4.6 µm, Q = 1.4–1.8(2.0) (Qm = 1.6 ± 0.1), ellipsoid to elongated-ellipsoid, in profile slightly curved or flattened on one side; in face view (3.7)4.0–5.0(5.1) µm broad, ellipsoid to elongated-ellipsoid, thin-walled, hyaline, non-amyloid. Basidia 24–34 × 5.6–7.9 µm, clavate, 2-spored, occasionally 4-spored. Pleurocystidia absent. Cheilocystidia 21–50 × 5.9–8.6 µm, abundant, slender cylindrical, occasionally fusiform, apex obtuse, occasionally mucronate or capitate, sometimes with small tubercles, thin-walled, hyaline. Pileipellis a cutis, hyphal cells 2.9–9.9 µm broad. Stipitipellis a cutis, hyphal cells 4.0–9.7 µm broad, thin-walled. Caulocystidia 29–100 × 5.1–8.4 µm, abundant, slender cylindrical, apex obtuse or capitate, thin-walled. Clamp connections present.

**Figure 2.**
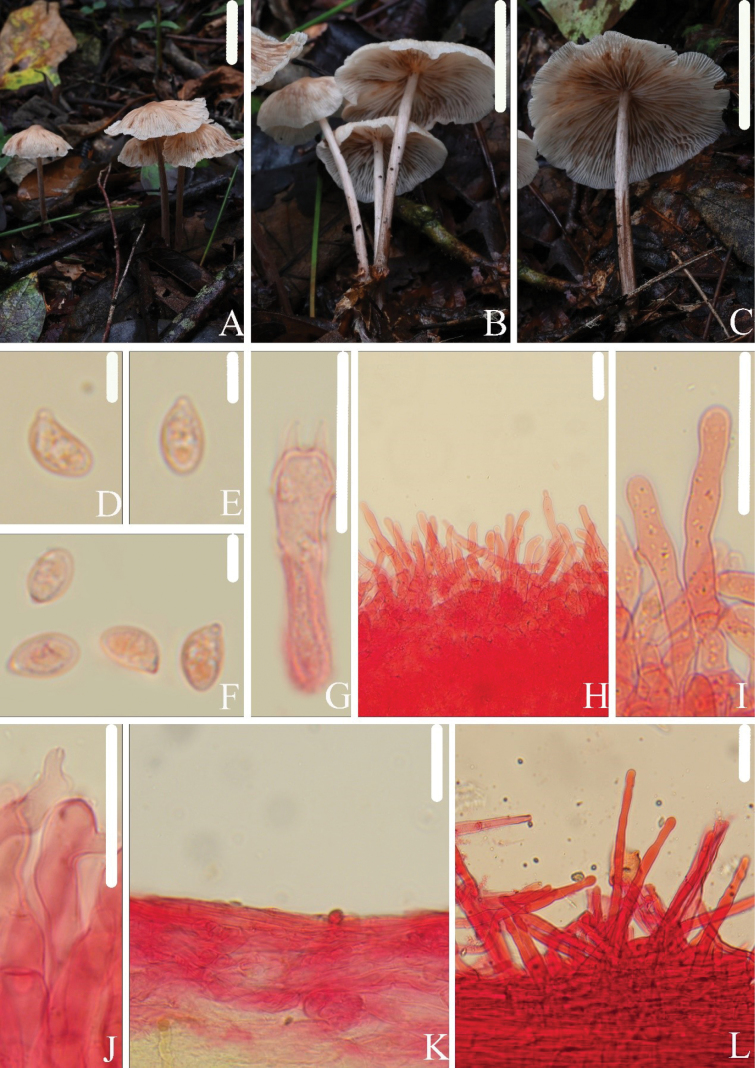
*Collybiopsissubpolygramma*. **A–C.** Basidiomata; **D–F.** Basidiospores; **G.** Basidia; **H–J.** Cheilocystidia; **K.** Pileipellis; **L.** Caulocystidia. All microscopic structures were observed in 5% KOH, and 1% Congo red was used as the stain. Scale bars: 30 mm (**A–C**); 5 µm (**D–F**); 20 µm (**G**); 30 µm (**H–I**); 20 µm (**J**); 30 µm (**K, L**).

##### Habitat.

Scattered or gregarious on the ground in mixed broad-leaved forests.

##### Distribution.

China.

##### Additional specimens examined.

China • Fujian Province: Wuyishan City, Daliankeng, 6 June 2022, Jun-Qing Yan and Lin-Gen Chen, HFJAU3551; Zhejiang Province: Lishui City, Qingtian County, Lanni Lake, 1 August 2021, Jun-Qing Yan, Bin-Rong Ke and Zhi-Heng Zeng, HFJAU2644; Qingtian, 31 August 2021, Ya-Ping Hu, HFJAU3277.

##### Remarks.

*Collybiopsissubpolygramma* is morphologically similar to *G.coracicolor* (Berk. & M.A. Curtis) J.L. Mata, *G.hondurensis* (Murrill) J.L. Mata, *C.neotropica* (Singer) R.H. Petersen and *C.polygramma*, all of which have a cutis of pileipellis without rameales-type structure, similarly shaped basidiospores, and the absence of pleurocystidia. However, *G.coracicolor* has smaller pileus (shorter than 25 mm), larger cheilocystidia (70 × 8–10 µm), and brown incrustation on the pileipellis hyphae (Dennis 1951). *Gymnopushondurensis* has four-spored basidia and clavate cheilocystidia (Mata and Ovrebo 2009). *Collybiopsisneotropica* has smaller pileus (shorter than 23 mm), larger basidiospores (8.2–9.7 × 4.3–4.8 µm), larger basidia (29–32 × 6.8–9.7 µm) with four spores, and irregular, filamentous cheilocystidia (Singer 1961). *Collybiopsispolygramma* has smaller pileus (shorter than 25 mm) with a distinct umbilicate, absence of cheilocystidia, and hyphae of stipe are thick-walled, up to 3 µm (Mata and Petersen 2003).

Additionally, *G.lodgeae* (Singer) J.L. Mata also has a cutis of pileipellis without rameales-type structure and similarly shaped basidiospores. However, *G.lodgeae* has a smaller pileus (shorter than 20 mm), larger cheilocystidia (32–56 × 10–14 µm), and the presence of pleurocystidia (Mata and Petersen 2003).

#### ﻿ Collybiopsis fucata

Taxon classificationFungiAgaricalesOmphalotaceae

J.Q. Yan, L. Ding, & S.N. Wang sp. nov.

9EE8397E-BF76-5649-82D1-C45836668700

858729

Fig. 3

##### Etymology.

Refer to the Latin “fucatus,” meaning “painted”—the lamellae edges are fucata.

##### Holotype.

China • Zhejiang Province, Lanni Lake, Qingtian County, Lishui City, 6 July 2021, Jun-Qing Yan, HFJAU2535.

##### Diagnosis.

This species is characterized by the reddish-brown, hygrophanous pileus; white lamellae that are adnate to adnexed; reddish-brown stipe with a white apex; basidiospores 3.9–5.2 µm broad; fusiform to utriform pleurocystidia; and cheilocystidia 25–65 × 11–31 µm.

##### Macrostructures.

Pileus 6.5–14 mm in diam., hemispherical when young, then becoming convex, applanate when mature, umbilicate at center, margin entire, surface smooth or with striations, hygrophanous, reddish-brown (8D4–5), with color fading from the center to the margin and gradually paling from the margin to the center as the hygrophanous effect disappears, center light brown (7D5–6). Lamellae adnate to slightly adnexed, distant, with 1–2 types of lamellulae, white, with entire edges, concolorous with the lamellae. Stipe 4.0–8.9 × 0.5–1.0 mm, central, hollow, cylindrical, equal, darkening towards the base, white at the apex to reddish-brown (8E5–6) at the base, surface covered with white fibrils, no white mycelium observed at the base.

**Figure 3.**
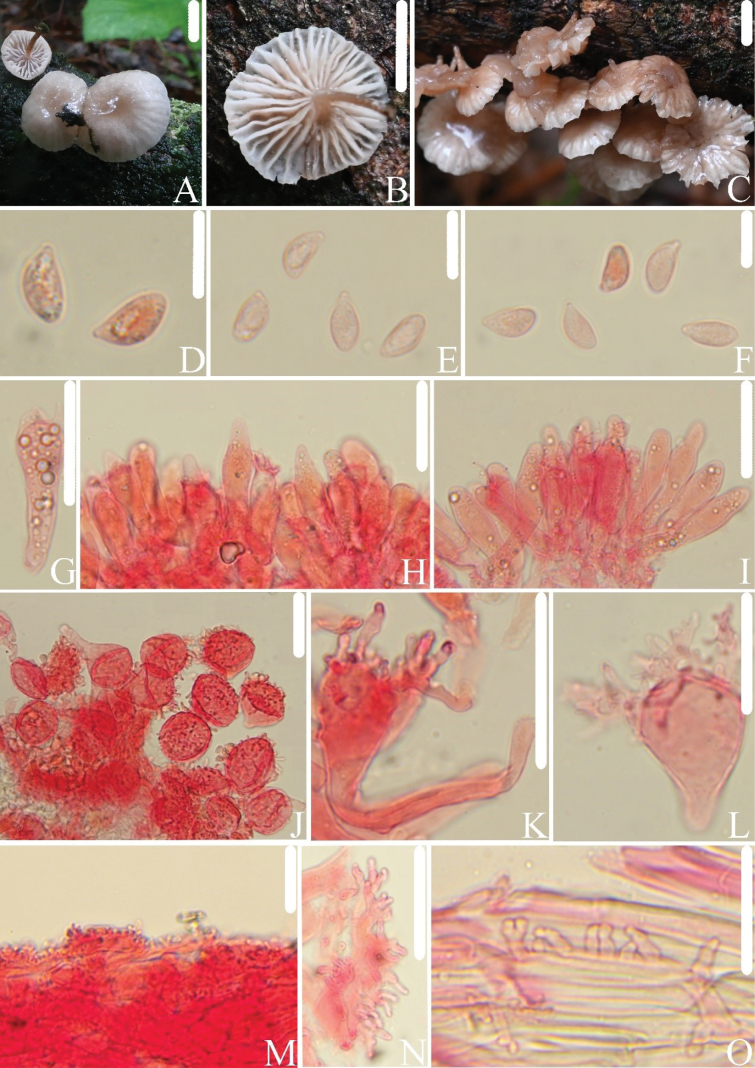
*Collybiopsisfucata*. **A–C.** Basidiomata; **D–F.** Basidiospores; **G.** Basidia; **H–I.** Pleurocystidia; **J–L.** Cheilocystidia; **M, N.** Pileipellis; **O.** Stipitipellis. All microscopic structures were observed in 5% KOH, and 1% Congo red was used as the stain. Scale bars: 5 mm (**A–C**); 10 µm (**D–F**); 20 µm (**G–I**); 30 µm (**J–O**).

##### Microstructures.

Basidiospores in side view [n = 118] (6.7)7.0–9.1(9.7) × (3.3)3.9–5.2(5.4) µm (av = 8.1 × 4.4 µm, Q = (1.5)1.6–2.1(2.2) (Qm = 1.8 ± 0.1), ellipsoid to elongated-ellipsoid, in profile slightly curved or flattened on one side; in face view 4.0–5.0(5.1) µm broad, ellipsoid to elongated-ellipsoid, thin-walled, hyaline, non-amyloid. Basidia 21–30 × 5.4–8.5 µm, clavate, 4- or 2-spored. Pleurocystidia 17–34 × 4.2–8.0 µm, abundant, fusiform, utriform, thin-walled, hyaline. Cheilocystidia 25–65 × 11–31 µm, abundant, with pedicellate spherical shapes, occasionally broad club-shaped or clavate, with short protuberances on the surface and occasionally branched, with pedicel 2.5–7.5 µm broad, branches 2.6–15 × 1.1–3.1 µm, thin-walled, hyaline. Pileipellis a cutis, with rameales-type structure, hyphal cells 2.1–8.6 µm broad. Stipitipellis a cutis, with numerous small protrusions on the hyphae, hyphal cells 2.9–13 µm broad, protrusions 3.2–13 × 1.5–6.1 µm, thin-walled. Clamp connections present.

##### Habitat.

Scattered or gregarious on decaying wood in mixed forests and broad-leaved forests.

##### Distribution.

China.

##### Additional specimens examined.

China • Hubei Province: Longmenhe Village, Xingshan County, Yichang City, 29 June 2024, Jun-Qing Yan, Lin-Gen Chen, Hong Chen, and Ling Ding, HFJAU5343; Zhejiang Province: Lanni Lake, Qingtian County, Lishui City, 1 August 2021, Jun-Qing Yan, Bin-Rong Ke and Zhi-Heng Zeng, HFJAU2653.

##### Remarks.

*Collybiopsisfucata* is morphologically similar to *C.californica* (Desjardin) R.H. Petersen, *C.filamentipes* R.H. Petersen, and *C.foliiphila* (A.K. Dutta, K. Acharya & Antonín) R.H. Petersen within the *C.ramealis* complex, all having a cutis of pileipellis with rameales-type structure and cheilocystidia with rameales-type structure. However, *C.californica* has a longer stipe (10–46 mm long), narrower basidiospores (3.0–3.3 µm), smaller cheilocystidia (20–42 × 6–20 µm), and absence of pleurocystidia (Desjardin 1987). *Collybiopsisfilamentipes* has adnexed to subdecurrent lamellae, a longer stipe (12–21 mm), narrower basidiospores (2.5–3.5 µm), and narrower cheilocystidia (8–18 µm broad), clavate to broad clavate, occasionally stoutly dichotomous (Petersen and Hughes 2021). *Collybiopsisfoliiphila* has white pileus, lamellae often with rusty tawny to dark brick-colored spots, narrower basidiospores (3.0–3.7 µm), smaller cheilocystidia (28.6–31 × 9–12 µm), and absence of pleurocystidia (Dutta et al. 2015).

#### ﻿ Collybiopsis dentata

Taxon classificationFungiAgaricalesOmphalotaceae

J.Q. Yan, L. Ding, & S.N. Wang sp. nov.

14566280-B2C8-5BFC-A7B4-7FFC0858F71A

858730

Fig. 4

##### Etymology.

Refer to the Latin “dens,” meaning “tooth”—the lamellae edges are dentate.

##### Holotype.

China • Zhejiang Province: Lishui City, Qingtian County, Shigu Lake, 31 July 2021, Jun-Qing Yan, Bin-Rong Ke, Zhi-Heng Zeng, HFJAU2586.

##### Diagnosis.

This species is characterized by white lamellae that are free, basidiospores 3.9–4.7 µm broad, fusiform to utriform pleurocystidia, and cheilocystidia measuring up to 30–71 × 17–29 µm.

##### Macrostructures.

Pileus 3.0–8.5 mm in diam., convex to plane, umbilicate, margin entire, sulcate towards margin, white, light brown (7D5–6) at the center. Lamellae free, distant, with 1–2 types of lamellulae, white, edges serrate, concolorous with the lamellae. Stipe 2.0–4.0 × 0.4–1.0 mm, central, hollow, cylindrical, equal, darkening towards the base, white at the apex to reddish-brown (8E5–6) at the base, surface covered with white pruinose, no white mycelium observed at the base.

**Figure 4.**
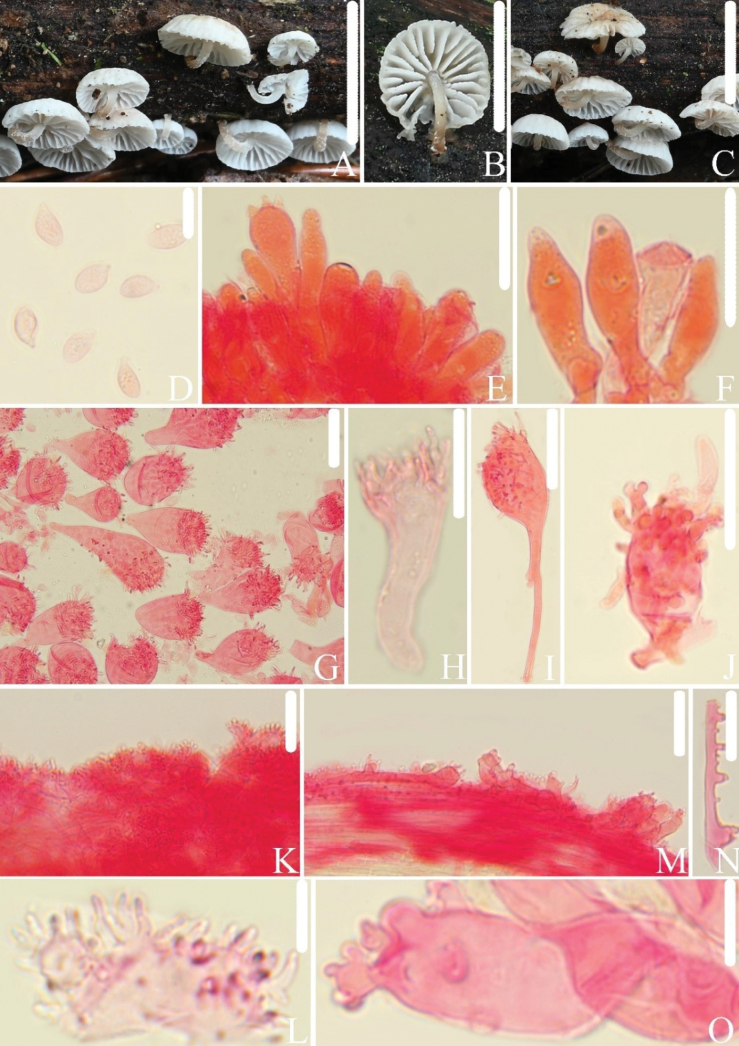
*Collybiopsisdentata*. **A–C.** Basidiomata HFJAU2586, Holotype; **D.** Basidiospore; **E, F.** Pleurocystidia; **G–J.** Cheilocystidia; **K, L.** Pileipellis; **M.** Stipitipellis; **N.** Stipe hyphae; **O.** Caulocystidia. All microscopic structures were observed in 5% KOH, and 1% Congo red was used as the stain. Scale bars: 10 mm (**A**); 5 mm (**B**); 10 mm (**C**); 10 µm (**D**); 20 µm (**E, F**); 30 µm (**G–K**); 10 µm (**L**); 30 µm (**M**); 10 µm (**N, O**).

##### Microstructures.

Basidiospores in side view [n = 44] (6.3)6.7–8.2(8.6) × (3.8)3.9–4.7(5.0) µm (av = 7.5 × 4.3 µm, Q = 1.6–2.0(2.1) (Qm = 1.7 ± 0.11), elongated-ellipsoid to cylindrical, in profile slightly curved or flattened on one side; in face view 3.9–4.9(5.0) µm broad, elongated-ellipsoid to cylindrical, thin-walled, hyaline, non-amyloid. Basidia 18–26 × 7.0–9.6 µm, clavate, 4- or 2-spored. Pleurocystidia 20–32 × 5.5–8.1 µm, abundant, fusiform, utriform, thin-walled, hyaline. Cheilocystidia (28)30–71(152) × 17–29 µm, abundant, pyriform, with pedicellate spherical shapes, broad clavate, with short projections on the surface, occasionally branched, with pedicel 2.6–7.9 µm broad, branches 3.2–7.6 × 0.6–1.7 µm, thin-walled, hyaline. Pileipellis a cutis, with rameales-type structure, hyphal cells 2.1–14 µm broad. Stipitipellis a cutis, with numerous small protrusions on the hyphae, hyphal cells 2.7–14 µm broad, protrusions 1.5–7.1 × 1.0–5.8 µm, thin-walled. Caulocystidia 8.8–55 × 5.1–14 µm, abundant, cylindrical, surface with protuberances, occasionally branches, branches 1.4–7.4 × 0.9–3.2 µm, thin-walled. Clamp connections present.

##### Habitat.

Gregarious on decaying wood in mixed forests.

##### Distribution.

China.

##### Additional specimens examined.

China • Zhejiang Province: Lishui City, Qingtian County, Shigu Lake, 31 July 2021, Jun-Qing Yan, Bin-Rong Ke, Zhi-Heng Zeng, HFJAU5718.

##### Remarks.

*Collybiopsisdentata* is morphologically similar to *C.californica*, *C.filamentipes*, and *C.foliiphila* within the *C.ramealis* complex, all having a cutis of pileipellis with rameales-type structure and cheilocystidia with rameales-type structure. However, *C.californica* has narrower basidiospores (3.0–3.3 µm), smaller cheilocystidia (20–42 × 6–20 µm), and absence of pleurocystidia (Desjardin 1987). *Collybiopsisfilamentipes* has adnexed to subdecurrent lamellae, narrower basidiospores (2.5–3.5 µm), narrower cheilocystidia (8–18 µm), smaller caulocystidia (5–20 × 6–9 µm), cylindrical to digitate, and rarely branched (Petersen and Hughes 2021). *Collybiopsisfoliiphila* has narrower basidiospores (3.0–3.7 µm), lamellae often with rusty tawny to dark brick-colored spots, smaller cheilocystidia (28.6–31 × 9–12 µm), and absence of pleurocystidia (Dutta et al. 2015).

#### ﻿ Collybiopsis latispora

Taxon classificationFungiAgaricalesOmphalotaceae

J.Q. Yan, L. Ding, & S.N. Wang sp. nov.

80C3ECCF-D74E-53C3-959D-53CD311479CA

858731

Fig. 5

##### Etymology.

Refer to the Latin “latus,” meaning “broad”—the species having wide basidiospores.

##### Holotype.

China • Fujian Province, Wuyishan Mountain, 12 August 2021, Qin Na, Yu-Peng Gai, HFJAU3109.

##### Diagnosis.

This species is characterized by white lamellae with a minute, snow-white ring or ruff at the stipe base; basidiospores 4.3–5.9 µm broad; subcylindrical, narrow fusiform, narrow utriform pleurocystidia; and cheilocystidia 16–47 × 13–24 µm.

##### Macrostructures.

Pileus 2.5–6.5 mm in diam., convex to plane, margin entire, sulcate towards margin, white, brownish-orange (7C3) at the center. Lamellae adnexed, distant, with 1–2 types of lamellulae, white, edges serrate, concolorous with the lamellae. Stipe 2.1–4.2 × 0.4–0.7 mm, central, hollow, cylindrical, equal, light brown (7D5–6), white at approximately three-quarters of the base, surface covered with white pruinose, with minute snow white ring or ruff on stipe base.

**Figure 5.**
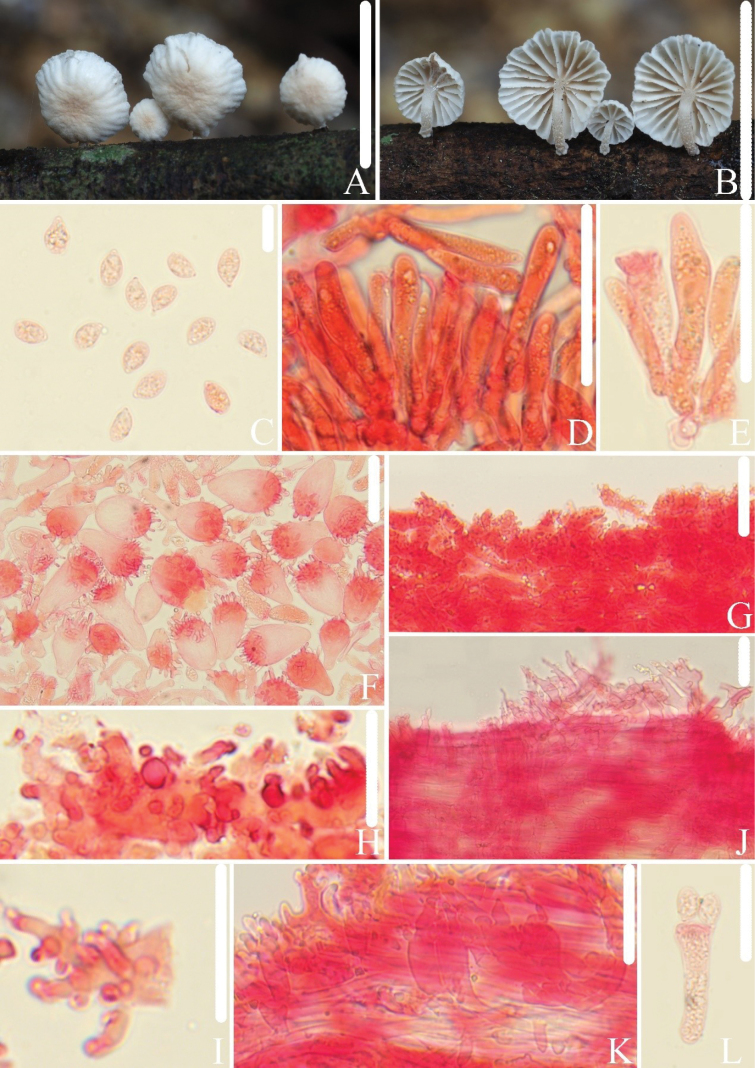
*Collybiopsislatispora*. **A, B.** Basidiomata; **C.** Basidiospores; **D, E.** Pleurocystidia; **F.** Cheilocystidia; **G–I.** Pileipellis; **J, K.** Stipitipellis and Caulocystidia; **L.** Basidia. All microscopic structures were observed in 5% KOH, and 1% Congo red was used as the stain. Scale bars: 10 mm (**A, B**); 10 µm (**C**); 30 µm (**D–G**); 20 µm (**H, I**); 30 µm (**J, K**); 20 µm (**L**).

##### Microstructures.

Basidiospores in side view [n = 43] (6.4)6.7–8.1(8.2) × (4.1)4.3–5.9(6.2) µm (av = 7.4 × 4.8 µm, Q = 1.3–1.6(1.8) (Qm = 1.5 ± 0.09), ellipsoid to elongated-ellipsoid, in profile slightly curved or flattened on one side; in face view (4.3)4.5–5.7(7.3) µm broad, broadly ellipsoid, ellipsoid to elongated-ellipsoid, thin-walled, hyaline, non-amyloid. Basidia 16–28 × 5.1–8.6 µm, clavate, 2- or 4-spored. Pleurocystidia 18–37 × 4.4–7.3 µm, abundant, subcylindrical, narrow fusiform, narrow utriform, thin-walled, hyaline. Cheilocystidia 16–47 × 13–24 µm, abundant, pyriform, broad clavate, with pedicellate spherical shapes, with short projections on the surface, occasionally branched, with pedicel 3.1–6.6 µm broad, branches 2.7–11 × 1.2–3.1 µm, thin-walled, hyaline. Pileipellis a cutis, with rameales-type structure, hyphal cells 3.8–9.1 µm broad. Stipitipellis a cutis, hyphal cells 3.3–7.7 µm broad, thin-walled. Caulocystidia 21–47 × 3.4–9.7 µm, abundant, cylindrical, surface with protuberances or branches, thin-walled. Clamp connections present.

##### Ecology.

Scattered on dead branches in mixed forests.

##### Remarks.

*Collybiopsislatispora* is morphologically similar to *C.californica*, *C.filamentipes*, and *C.foliiphila* within the *C.ramealis* complex, all having a cutis of pileipellis with rameales-type structure and cheilocystidia with rameales-type structure. However, *C.californica* has a longer stipe (10–46 mm), narrower basidiospores (3.0–3.3 µm), and absence of pleurocystidia (Desjardin 1987). *Collybiopsisfilamentipes* has a longer stipe (12–21 mm), smooth, narrower basidiospores (2.5–3.5 µm), and cheilocystidia that are typically clavate to broad clavate, occasionally stoutly dichotomous (Petersen and Hughes 2021). *Collybiopsisfoliiphila* has lamellae often with rusty tawny to dark brick-colored spots, narrower basidiospores (3.0–3.7 µm), narrower cheilocystidia (9–12 µm), and absence of pleurocystidia (Dutta et al. 2015).

## ﻿Discussion

Molecularly and morphologically, *C.fucata*, *C.latispora*, and *C.dentata* all belong to the *C.ramealis* complex. In a previous study, this complex included five species, namely *C.foliiphila*, *C.filamentipes*, *C.californica*, *C.furtiva* R.H. Petersen and *C.ramealis* (Petersen and Hughes 2021). The morphological differences between *C.californica*, *C.filamentipes*, *C.foliiphila*, and the new species have been discussed in Remarks. The differences between *C.furtiva*, *C.ramealis*, and the three new species are as follows: *C.furtiva* has narrower basidiospores (2.5–3.0 µm), only fusiform pleurocystidia, narrower cheilocystidia (10–13 µm), adnexed to decurrent lamellae, and fimbriate lamellar edges (Petersen and Hughes 2021). *Collybiopsisramealis* has a smooth stipe, subdecurrent lamellae, narrower basidiospores (2.5–4.0 µm), and digitate to narrow fusiform pleurocystidia (Petersen and Hughes 2021). In addition, *C.humillima* (Quél.) Argaud conforms to the complex both morphologically and molecularly. However, *C.humillima* has smaller pileus (shorter than 3.5 mm), narrower basidiospores (2.7–3.0 µm), narrower cheilocystidia (6.5–11.5 µm), and only clavate cheilocystidia, which can be clearly distinguished from the three new species (Noordeloos 1983).

*Collybiopsisfucata* and *C.latispora* formed a sister lineage, but they can be easily distinguished by morphologies. The former has a hygrophanous, reddish-brown pileus; larger cheilocystidia (25–65 × 11–31 µm) with denser surface protuberances; and no minute, snow white ring or ruff on the stipe base. The latter has a non-hygrophanous, white pileus; smaller cheilocystidia (16–47 × 13–24 µm) with sparser surface protuberances; and with a minute snow white ring or ruff on the stipe base.

*Collybiopsisdentata* formed a stable branch on its own. It differs from *C.fucata* by its white, non-hygrophanous pileus, free lamellae, and serrated lamellar edges. And distinguished from *C.latispora* by the absence of a minute snow white ring or ruff on the stipe base, larger cheilocystidia (30–71 × 17–29 µm), and denser surface protuberances.

The major species of wood-inhabiting fungi are in Polyporales and Hymenochaetales, and extensive and systematic studies on these two orders have been carried out in China (Dai 2010; Cui et al. 2019; Dai et al. 2021; Wu et al. 2022a, 2022b; Wang et al. 2024; Liu et al. 2025b, 2025c). However, a few reports on wood-inhabiting fungi in Agaricales were published (Zhang et al. 2023a; Liu et al. 2024; Liu et al. 2025a), so the species diversity of wood-inhabiting Agaricales is still poorly known. Our present study expands our understanding of *Collybiopsis* species by providing descriptions and phylogenetic analyses for the four new species. These findings enrich our knowledge of the diversity of *Collybiopsis* in China. As more investigations on wood-inhabiting Agaricales are carried out, more species of *Collybiopsis* will be found.

### ﻿Key to morphologically similar species

**Table:** 

1	Pileipellis composed of cylindrical, often sub-inflated hyphae, not a rameales-type structure	**2**
–	Pileipellis composed of a coarse rameales-type structure hyphae	**7**
2	Pleurocystidia present	***G.lodgeae***
–	Pleurocystidia absent	**3**
3	Pileus hyphae encrusted with brownish granules	***G.coracicolor***
–	Pileus hyphae not encrusted with brownish granules	**4**
4	Cheilocystidia absent	***C.polygramma***
–	Cheilocystidia present	**5**
5	Pileus 32–61 mm in diameter	***C.subpolygramma***
–	Pileus < 25 mm in diameter	**6**
6	Cheilocystidia narrowly clavate to clavate or broadly clavate to narrowly sphaero-pedunculate, some subfusoid to fusoid; apex obtuse, mucronate, diverticulate, or knobbed	***G.hondurensis***
–	Cheilocystidia filamentous, irregular, sometimes thickened or bulbous at the base, or thickened at the apex, and often constricted in multiple places	***C.neotropica***
7	Basidiospores > 4.0 µm wide	**8**
–	Basidiospores < 4.0 µm wide	**10**
8	Pileus hygrophanous, reddish-brown	***C.fucata***
–	Pileus not hygrophanous, white	**9**
9	Pleurocystidia subcylindrical, narrow fusiform, narrow utriform, the branches of the cheilocystidia 2.7–11 × 1.2–3.1 µm, basidiospores 6.7–8.1 × 4.3–5.9 µm, ellipsoid to elongated-ellipsoid, and with a minute snow white ring or ruff on the stipe base	***C.latispora***
–	Pleurocystidia fusiform, utriform, the branches of the cheilocystidia 3.2–7.6 × 0.6–1.7 µm, basidiospores 6.7–8.2 × 3.9–4.7 µm, elongated-ellipsoid to cylindrical, no minute snow white ring or ruff on stipe base	***C.dentata***
10	Minute snow white ring or ruff on stipe base	***C.furtiva***
–	No minute snow white ring or ruff on stipe base	**11**
11	Lamellae with flocculose, stipe fauve to brownish	***C.humillima***
–	Lamellae not with flocculose, stipe not fauve to brownish	**12**
12	Pleurocystidia absent	**13**
–	Pleurocystidia present	**14**
13	Lamellae buff to pale pinkish buff, appearing whitish-crystalline when dried; stipe apex buff, central portion rusty red, base reddish-brown	***C.californica***
–	Lamellae white, often with rusty tawny to dark brick-colored spots; stipe greyish yellow to buff-brown; overall stipe turns rusty tawny to brick upon drying	***C.foliiphila***
14	Lamellae subdistant to close (40–43), subdecurrent, adnexed to adnate to appearing free, basidiospores slender ellipsoid, pleurocystidia digitate to narrowly fusiform	***C.ramealis***
–	Lamellae few (12–13), adnexed to subdecurrent, basidiospores cylindrical to elongate-ellipsoid, pleurocystidia stalked-fusiform, rounded apically	***C.filamentipes***

## Supplementary Material

XML Treatment for Collybiopsis subpolygramma

XML Treatment for Collybiopsis fucata

XML Treatment for Collybiopsis dentata

XML Treatment for Collybiopsis latispora

## ﻿Additional information

### Author ORCIDs

Ling Ding https://orcid.org/0009-0000-5293-046X

Ben-Jian Zhong https://orcid.org/0009-0004-0817-9161

Hui Zeng https://orcid.org/0000-0003-2025-844X

Sheng-Nan Wang https://orcid.org/0000-0003-0648-271X

Jun-Qing Yan https://orcid.org/0000-0003-1128-5171

### Data availability

All of the data that support the findings of this study are available in the main text.
